# Phylogenomic analyses and comparative genomics of *Pseudomonas syringae* associated with almond (*Prunus dulcis*) in California

**DOI:** 10.1371/journal.pone.0297867

**Published:** 2024-04-11

**Authors:** Tawanda E. Maguvu, Rosa J. Frias, Alejandro I. Hernandez-Rosas, Brent A. Holtz, Franz J. A. Niederholzer, Roger A. Duncan, Mohammad A. Yaghmour, Catherine M. Culumber, Phoebe E. Gordon, Flavia C. F. Vieira, Philippe E. Rolshausen, James E. Adaskaveg, Lindsey P. Burbank, Steven E. Lindow, Florent P. Trouillas

**Affiliations:** 1 Department of Plant Pathology, University of California, Davis, Davis, CA, United States of America; 2 Kearney Agricultural Research and Extension Center, Parlier, CA, United States of America; 3 University of California Cooperative Extension, CA, United States of America; 4 Department of Plant Pathology and Microbiology, University of California, Riverside, Riverside, CA, United States of America; 5 U.S. Department of Agriculture, Agricultural Research Service, Parlier, CA, United States of America; 6 Department of Plant and Microbial Biology, University of California, Berkeley, Berkeley, CA, United States of America; University of Wisconsin Milwaukee, UNITED STATES

## Abstract

We sequenced and comprehensively analysed the genomic architecture of 98 fluorescent pseudomonads isolated from different symptomatic and asymptomatic tissues of almond and a few other *Prunus* spp. Phylogenomic analyses, genome mining, field pathogenicity tests, and *in vitro* ice nucleation and antibiotic sensitivity tests were integrated to improve knowledge of the biology and management of bacterial blast and bacterial canker of almond. We identified *Pseudomonas syringae* pv. *syringae*, *P. cerasi*, and *P. viridiflava* as almond canker pathogens. *P. syringae* pv. *syringae* caused both canker and foliar (blast) symptoms. In contrast, *P. cerasi* and *P. viridiflava* only caused cankers, and *P. viridiflava* appeared to be a weak pathogen of almond. Isolates belonging to *P. syringae* pv. *syringae* were the most frequently isolated among the pathogenic species/pathovars, composing 75% of all pathogenic isolates. *P. cerasi* and *P. viridiflava* isolates composed 8.3 and 16.7% of the pathogenic isolates, respectively. Laboratory leaf infiltration bioassays produced results distinct from experiments in the field with both *P. cerasi* and *P. syringae* pv. *syringae*, causing significant necrosis and browning of detached leaves, whereas *P. viridiflava* conferred moderate effects. Genome mining revealed the absence of key epiphytic fitness-related genes in *P. cerasi* and *P. viridiflava* genomic sequences, which could explain the contrasting field and laboratory bioassay results. *P. syringae* pv. *syringae* and *P. cerasi* isolates harboured the ice nucleation protein, which correlated with the ice nucleation phenotype. Results of sensitivity tests to copper and kasugamycin showed a strong linkage to putative resistance genes. Isolates harbouring the *ctpV* gene showed resistance to copper up to 600 μg/ml. In contrast, isolates without the *ctpV* gene could not grow on nutrient agar amended with 200 μg/ml copper, suggesting *ctpV* can be used to phenotype copper resistance. All isolates were sensitive to kasugamycin at the label-recommended rate of 100μg/ml.

## Introduction

Bacterial canker and bacterial blast of almond (*Prunus dulcis*) are two phases of a disease that can affect many parts of the almond tree, including flowers, leaves, trunks, and scaffold branches. Bacterial canker generally affects trees that are stressed or predisposed by biotic and abiotic factors, such as poor fertilization and nutritional status of trees, susceptible rootstocks, cold and humid environments, and a high population of ring nematodes associated with sandy soils [1, 2]. Bacterial canker is characterized by small to large irregular cankers with amber-colored gum on trunks that can lead to tree death. During cold, wet conditions in the spring, flowers and developing leaflets may become infected and give rise to the blast phase of the disease. A major predisposing factor for bacterial blast is freezing temperatures at bloom time. Bacterial blast causes flowers to turn dark brown and wither suddenly. Blast has been particularly severe in the past few years (2019 to 2021 and 2023) in almond orchards in the Sacramento and San Joaquin valleys of California.

In California, bacterial blast and canker has been mainly attributed to *Pseudomonas syringae* pv. *syringae*, although few studies have attempted to comprehensively characterize *Pseudomonas* species affecting almond. The plant genus *Prunus* comprises a group of hosts associated with a larger number of polyphyletic pathovars and species within the *P. syringae* species complex compared to other known host plants of *P. syringae* [3]. Traditionally, *P. syringae* isolates have been characterized using phenotypic and biochemical methods, sometimes complemented with amplification of the phytotoxin-encoding genes [4]. Although these methods have been used successfully in taxonomy, they have several limitations. For example, the phytotoxins used for the identification of *P. syringae* pv. *syringae* are not restricted to this pathovar or this species as it is common in isolates of the pathovars *aptata* and *atrofaciens* as well as in isolates of *P. fuscovaginae* [4]. Moreover, considering the plasticity of taxonomically relevant phenotypes between and within closely related isolates of the *P. syringae* species complex [5, 6], and the reported emergence of hybrid phylogroups through homologous recombination and horizontal gene transfer [7], robust identification methods should be used to ascertain the identity of isolates. Precise species/isolate identification has important implications in disease diagnosis, which in turn influences management strategies. Whole genome sequencing-based phylogenomic analyses have greatly improved the identification of species, as they elucidate the functional profiles of taxonomic groups and resolve ambiguities in the phylogeny of higher taxa that is rather difficult using traditional approaches [8, 9]. To improve the diagnostics and management of bacterial canker and bacterial blast of almond, comprehensive whole genome-based phylogenomic, pathogenesis, and resistance/susceptibility studies of almond-pathogenic *Pseudomonas* species is crucial.

*P. syringae* pv. *syringae* belongs to the *P. syringae* species complex, a complex which forms a monophyletic group within the *P. fluorescens*-like major branch of the *Pseudomonas* genus [10]. The complex is divided into nine genomospecies based on the whole genome phylogenomic studies, 13 phylogroups defined by multi-locus sequence analysis, and about 60 pathovars based on pathogenic characters [11–13]. Members of the species complex exhibit a variety of interactions with plants ranging from benign commensal associations, and opportunistic phytopathogens, to host specialized phytopathogens [7, 14]. Although individual strains are reported to show host genotype specificity [15], this is not always the case as several polyphyletic pathovars can infect a single species, whereas some strains affect a variety of crops [5, 16].

What makes some members of the *P. syringae* species complex successful phytopathogens has been well established. They carry a type III secretion system (T3SS), a key virulent determinant, that acts like a molecular syringe to translocate a suite of T3SS effector proteins (T3Es) into plant cells, as a primary mechanism of pathogenicity [17, 18]. The *P. syringae* T3E effectors have a myriad of functions some of which include antagonizing the plant’s innate immunity through activating jasmonate (JA) signaling through direct interactions with JAZ repressor proteins [19, 20]. They also suppress pathogen-associated molecular patterns (PAMP)-induced oxidative burst and stomatal closure [21, 22]. Detailed information about the structure, variation, mode of action, and distribution of T3SS among *P. syringae* phylogroups was provided recently [23]. The T3SS is not the sole determinant of virulence and host range for *P. syringae*; coordination of host physiological responses and metabolic pathways is also necessary for pathogen growth within host tissue [24]. *P. syringae* species have been shown to produce several phytotoxins that can be co-ordinately regulated by T3SS to aid pathogenesis, albeit secreted independently from T3SS [25]. For example, the coronatine toxin produced by some *P. syringae* isolates has been shown to promote virulence in plants by activating a signaling cascade that inhibits salicylic acid accumulation [26]. Other toxins produced by *P. syringae* include phaseolotoxin, mangotoxin, and tabtoxin which interfere with the plant ammino acid biosynthesis pathway [27–29]. Toxins like syringomycin and syringopeptin have been shown to have membrane disruption and ion-leakage activities [30]. Besides the toxins and T3E, several genes within the functional categories of amino acid and polysaccharide biosynthesis have been shown to contribute significantly to the success of *P. syringae* during both epiphytic and endophytic phases [31]. It is important to note that several factors including weather and host susceptibility are also essential for pathogenesis. For comprehensive details of the features contributing to the successful pathogenesis of *P. syringae* refer to [23].

In this study, we used next-generation sequencing to perform a comprehensive phylogenomic analysis of 98 fluorescent pseudomonad bacteria isolated from symptomatic and asymptomatic almond tissues. We incorporated several bioinformatics tools to identify virulence and antibiotic resistance determinants associated with the isolates. Virulence and antibiotic resistance genotypic data was complemented with the phenotypes of ice nucleation ability, pathogenicity, and bactericide/antibiotic resistance. The pathogenicity tests included field and laboratory assays, which showed distinct but explainable results. From this study, we comprehensively identified those *Pseudomonas* species pathogenic to almonds and designed PCR primers for *in planta* diagnosis. This work will facilitate future epidemiological studies, modelling for disease outbreak and predictions, and understanding pathogen-commensals-host interaction. Based on our findings, we provide recommendations for the best management practices using the currently available options in California.

## Materials and methods

### Sample isolation, DNA extraction, and whole genome sequencing

During the spring and fall of 2021 and winter and spring of 2022, 880 isolates of *Pseudomonas* spp. were isolated from symptomatic and asymptomatic flowers, dormant vegetative and flower buds, leaves, twigs, and bark tissues of almond, and a few other *Prunus* species in approximately 60 orchards in the Sacramento and San Joaquin valleys of California. A1 in S1 Data provides detailed information on the isolates’ metadata. Sampled tissues were shaken in 20 ml phosphate-buffered saline (PBS) with 0.02% Tween 20 for 30 minutes using an orbital shaker, and 100 μl of the resulting suspension was plated onto King’s B (KB) medium containing 50 μg/ml of cycloheximide. Putative *Pseudomonas* isolates were selected based on their fluorescence and sub-cultured twice to obtain pure cultures. All isolates are stored at -80°C in our culture collection at the Kearney Agricultural Research and Extension Center (KARE). A total of 98 isolates were selected for sequencing, 51 from symptomatic tissues and 47 from asymptomatic tissues (A1 in S1 Data). Among the isolates sequenced, 13 were obtained from stone fruit trees including apricot, cherry and plum (A1 in S1 Data). An attempt was made to be inclusive of the diversity of putative *Pseudomonas* strains by collecting colonies that exhibited fluorescence on KB media, irrespective of colony appearance. Sequencing of the 16S rRNA was used to prescreen the isolates for whole genome sequencing. Genomic DNA of the selected isolates was extracted and purified using the FastDNA^®^ SPIN Kit (MP Biomedicals, Irvine, CA) following the manufacturer’s protocol. Agarose gel electrophoresis and NanoDrop spectrophotometry (ND-100, NanoDrop Technologies Inc, Wilmington, DE, USA) were used to determine the integrity and purity of the resultant DNA, respectively. The DNA was submitted for whole genome sequencing to the Microbial Genome Sequencing Center (MIGS, Pittsburgh, PA), and paired-end reads (2x151bp) were generated from Illumina sequencing.

### Genome assembly, and annotations

Raw sequence reads were quality-filtered using FastQC (Babraham Bioinformatics - FastQC A Quality Control tool for High Throughput Sequence Data), and trimming was performed using Trimmomatic [32]. *De novo* assembling of the quality reads was performed using SPAdes version 3.13.0 [33]. CheckM [34] was used to assess the quality of the assembled genomes. SPAdes assembled genomes were annotated by RAST [35] with default settings. The analysis was performed on the Kbase platform [36]. Based on CheckM results, genomes with ≤ 2% contamination were used for downstream analyses. Resistance Gene Identifier (RGI) was utilized to predict resistomes in the genomic sequences using homology and SNP models on the Comprehensive Antibiotic Resistance Database with default settings [37], and the Virulence Factors of Pathogenic Bacteria database [38] was used to identify virulence factors. Secondary metabolite biosynthetic gene clusters (BGC) were predicted using the antiSMASH bacterial version [39].

### Whole genome-based phylogenomic analysis

The SPAdes-assembled genomes were uploaded to the Type Strain Genome Server (TYGS) [40]. A pairwise comparison of the user-uploaded genomes and the phylogenetically related type strains was performed using Genome Blast Distance Phylogeny (GBDP). Inter-genomic distances were inferred using the trimming algorithm and distance formula *d*_*5*_, with 100 replicates [41]. Digital DNA-to-DNA hybridization (dDDH) values and confidence intervals were calculated using the recommended settings of the Genome-to-Genome Distance Calculator (GGDC) 2.1. The resulting inter-genomic distances were used to infer a balanced minimum evolutionary tree with branch support via FASTME 2.1.4, including SPR post-processing [42]. Trees were rooted at the midpoint and visualized with PhyD3 [43]. Pairwise genome comparisons based on average nucleotide identity (ANIb) were done using JSpecies software [44]. The genome taxonomy database toolkit (GTDB-Tk) was also used to assign taxonomy based on the genome taxonomy database [45]. To generate a finalized phylogenetic tree, core genome alignments of the RAST annotated genomes were used to infer a whole genome based approximately maximum likely wood phylogenetic tree using FastTree version 2.1.10 [46]. The analysis included at least one representative genome sequence of the established *P. syringae* phylogroups when a genome sequence could be attained publicly. The resulting tree was imported to the interactive tree of life [47] for display and adding annotation features. The orthovenn3 server for whole genome comparison and annotation of orthologous clusters was used to compare the proteome among the isolates [48].

### Pathogenicity tests

Based on the phylogenomic analysis, 26 isolates representing the various designated species complexes were selected for pathogenicity testing, with a bias towards members of the *P. syringae* species complex. To ensure a complete representation, at least two isolates from each designated clade or species complex were included in the pathogenicity studies. Bacterial suspensions of each selected isolate were prepared from 30-h-old KB colonies in 12 ml Falcon tubes containing sterile phosphate-buffered saline (PBS). The inoculum concentration was adjusted to approx. 10^8^ CFU/ml (90% transmission at OD600) using a DU730 Life Sciences UV/Vis Spectrophotometer (Beckman Coulter Inc., Fullerton, CA, USA). A similar procedure was used for all other inoculation experiments.

For the canker field experiments at Kearney Agricultural Research and Extension Centre (KARE), the inoculation sites on 2- to 3-year-old *P. dulcis* cv. Nonpareil branches were first disinfected with 90% ethanol in Nov 2022, Feb 2023, and Apr 2023. A sterile nail was then used to puncture the bark to create 3 wounds/branch/tree for each isolate. The experiment was laid out in four 4-tree blocks with each isolate contained in each block, resulting in a total of four tree replicates per isolate. Bacterial suspensions were sprayed onto the wounds using a mist spray bottle, and PBS was used as a control. Disease evaluation was done after 4 months. To determine the isolates’ virulence, the bark was removed with a knife, and canker lengths were measured. To fulfill Koch’s postulates, active canker margins were plated onto KB medium, and isolated fluorescent bacteria were re-streaked to obtain pure colonies. The identity of the recovered isolates was determined by PCR using primers we designed for the diagnosis of the pathogenic strains (A7 in S1 Data). PCR conditions are specified in A8 in S1 Data.

For leaf pathogenicity tests (blast phase) in the field, flowers, and leaflets of cv. Nonpareil almond at KARE were inoculated on Feb 22, 2023. Nine to ten approximately 12- to 14-cm-long flowering shoots were used for each treatment, and PBS was used as a control. This experiment was laid out in six block replicates. Shoots were spray-inoculated as described above for cankers, and disease was evaluated after two weeks. For this, the first 15 leaves from the tip of each shoot were assessed for necroses and leaf spots (A6 in S1 Data). The incidence of disease was determined based on the number of leaves with blast symptoms on the 15 leaves examined for each replication of each isolate. In addition, the severity of disease was measured based on a score of 0 = intact leaves with no visible necrosis or spots, 1 = leaves with marginal necrosis, 2 = leaves with marginal necrosis and spots covering <50% of the blades, and 3 = leaves with marginal necrosis and spots covering at >50% of the blades. This experiment was done twice.

For inoculations of detached leaves, freshly collected cv. Nonpareil leaves were washed under running tap water for 5 min, dipped into 70% ethanol for 1 min, rinsed with sterile distilled water, and then dipped into 6% sodium hypochlorite for 5 min. The leaves were then rinsed four times with sterile distilled water and air-dried in a sterile flow bench. A pressure infiltration method was used to inoculate the leaves on the abaxial side using sterile 1-ml disposable syringes without a needle. Gentle pressure was applied until the mesophyll tissue appeared water-soaked. For each isolate, four leaves were inoculated. PBS was used as a control. The leaves were placed on a raised plastic mesh inside a transparent crisper box containing paper towels wetted with sterile distilled water and incubated at 25°C under natural light conditions. Data were recorded after 5 days. This experiment was done twice. For all the pathogenicity tests, mean comparison for lesion length between control and pseudomonas treatments was made by unpaired t-test. Results are reported as mean ± standard error of the mean, unless otherwise stated. For comparison among groups, analysis of variance (ANOVA) was used.

### Ice nucleation assay

Ice nucleation tests on 20 isolates, representative of each phylogenomic species within the designated *P. syringae* species complex, were done following the method described by Lindow et al. [49].

### Kasugamycin and copper sensitivity tests

Isolates of the identified pathogenic species (*P. syringae* pv. *syringae*, *P. cerasi* and *P. viridiflava*) were tested for their sensitivity to kasugamycin and copper, two bactericides used to manage bacterial blast in California. All *P. cerasi* (2) and *P viridiflava* (4) isolates were used for kasugamycin and copper susceptibility tests. For *P. syringae* pv. *syringae*, a total of six isolates were used, 3 with the *ctpV* gene and 3 without the *ctpV* gene. Two isolates for *P. viridiflava* also possessed the *ctpV* gene. To evaluate the toxicity of kasugamycin (Kasumin 2L, Arysta LifeScience, Cary, NC), the agar dilution plate method was used. Nutrient agar was amended with 100 μg/ml kasugamycin, the labeled rate of the product. Unamended plates were used as a control. For copper (Champ® WG, Nufarm Americas Inc) nutrient agar was amended with 100, 200, 300, 400, 500 or 600 μg/ml metallic copper equivalent (MCE). Aliquots of 2 μl bacterial suspensions at 10^8^ CFU were inoculated onto control and bactericide-amended plates. Plates were incubated for 48 h at 25°C, and visually inspected for bacterial growth. For kasugamycin, growth on amended plates was considered resistance. For copper, resistance was defined as visible growth at concentrations that inhibited growth of the other isolates. The experiment was done three times.

### Primer design for the identified pathogenic species

Primers were designed to target genes encoding the T3SS effector proteins HrpR of *P. viridiflava* or AvrE of *P. cerasi* and *P. syringae* pv. *syringae* using Primer-BLAST (https://www.ncbi.nlm.nih.gov/tools/primer-blast/) and their specificity was confirmed in PCR assays using target and non-target isolates. The designed primers were tested for their suitability for real-time PCR using 10-fold serial dilutions of the target organism DNA. We also exploited variations in these genomic sequences to ensure primer specificity at the species level. We also designed primers that target the *ctpV* gene, a gene that confer resistance to copper.

No permit was required for this work, *P. syringae* pv. *syringae* is not a quarantine pathogen in California.

## Results and discussion

### Phylogenomic analyses

**Fig 1:**
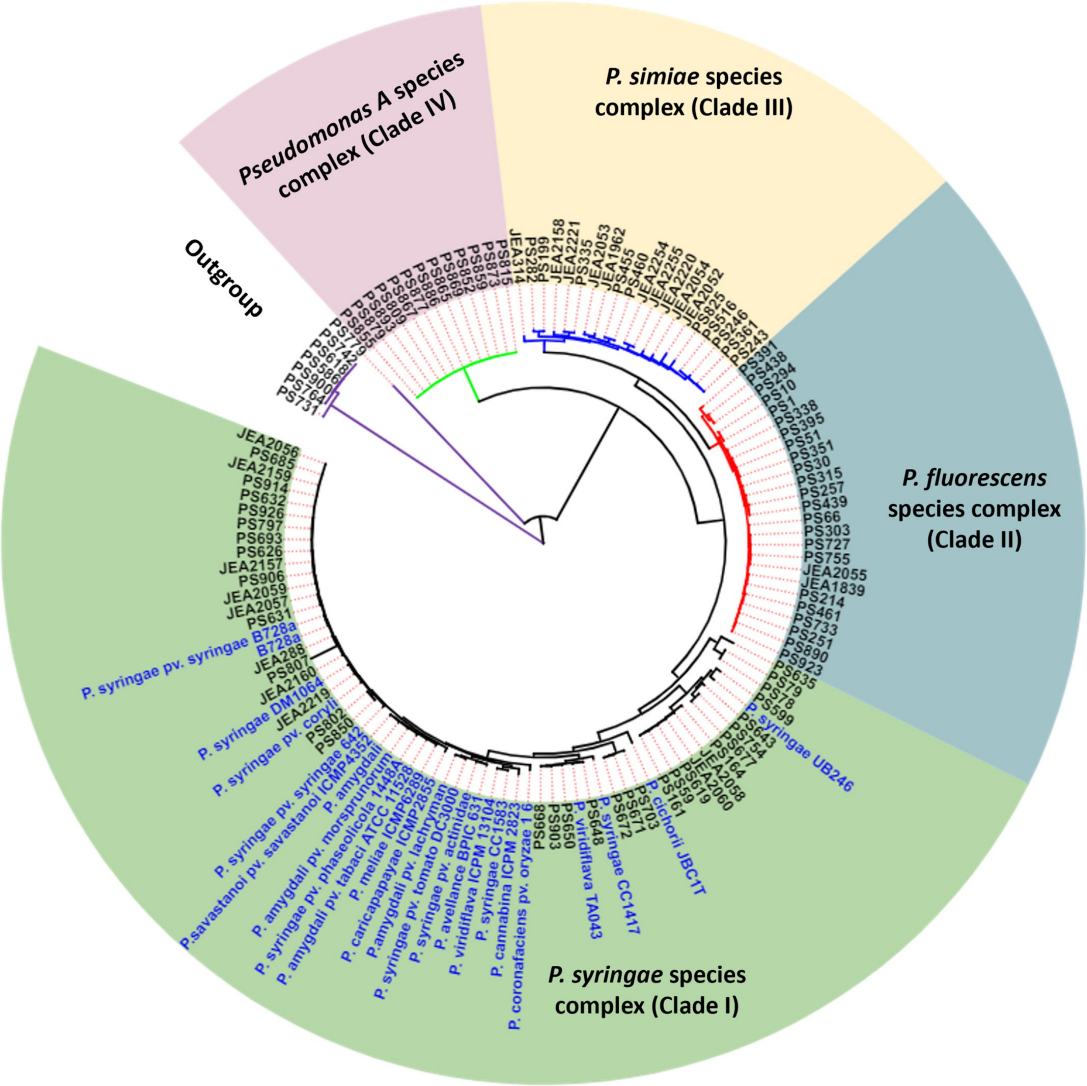
Whole genome sequence-based phylogenomic analysis of pseudomonads associated with almonds in California. Phylogenomic analyses established four distinct clades that were designated species complexes of *P. syringae* (clade I), *P. fluorescens* (clade II), *P. simiae* (clade III), and *Pseudomonas A* (clade IV). The outgroup consists of non-pseudomonads used to benchmark the analysis.

**Fig 2:**
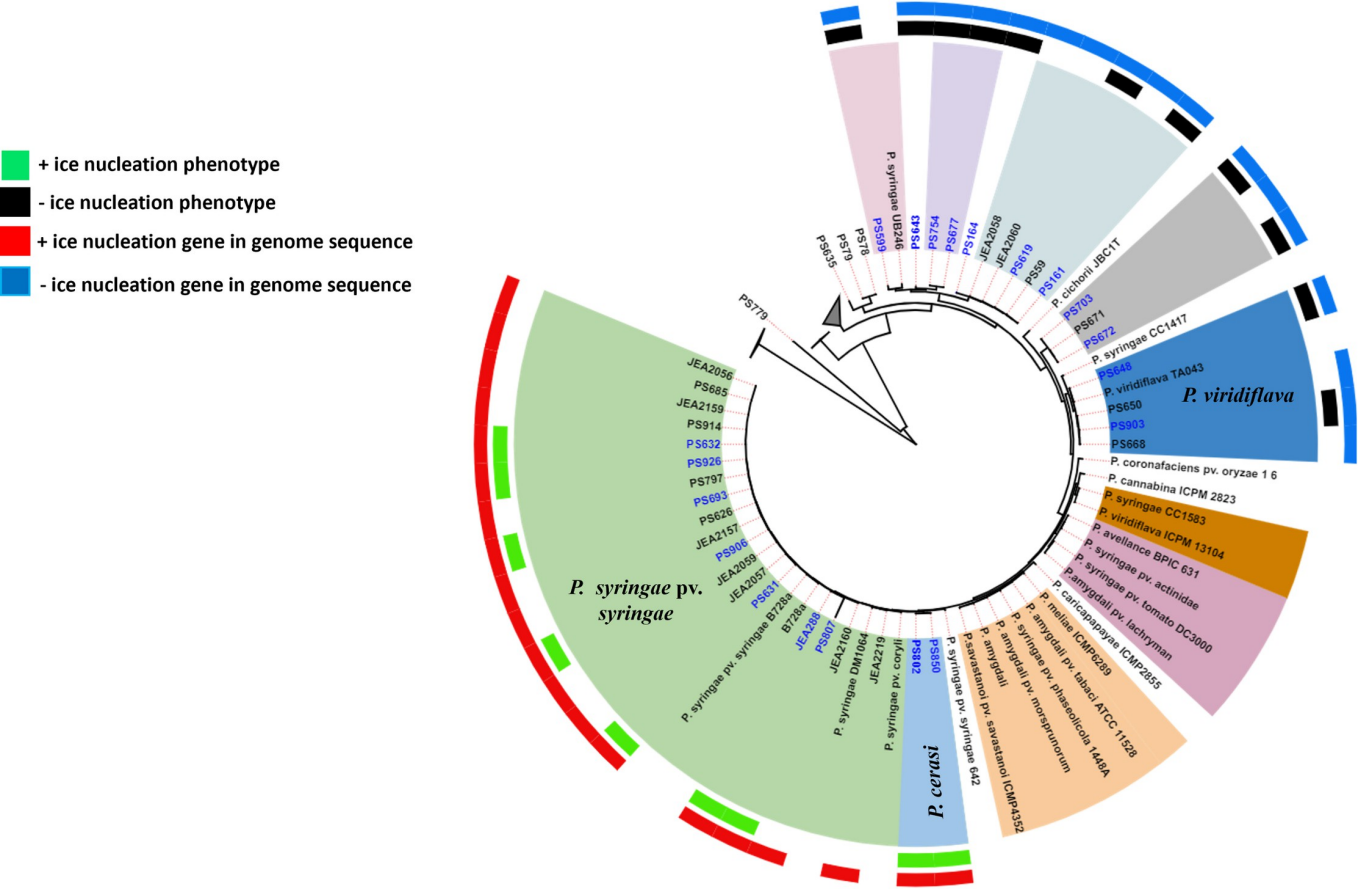
A close-up of the phylogenomic analysis of the *P. syringae* species complex (clade I) from Fig 1. The different species within the *P. syringae* species complex are shown. The isolates with codes in blue were subjected to a phenotypic ice nucleation test. Isolates from this study have the prefix (PS) or (JEA), all the other isolates are reference strains with sequences obtained from the NCBI database. The isolates we obtained from almonds in California were taxonomically classified as *P. syringae* pv. *syringae* (I), *P. cerasi* (II) and *P. viridiflava* (III). Isolates from this study that did not form a species cluster with established members of the *P. syringae* species complex were presumed to be putative new species and they are referred to as *P. syringae* throughout the manuscript. Taxonomic classification was based on digital DNA-DNA hybridization (dDDH) and average nucleotide identity (ANIb) that were in congruence.

Quality metrics for the assembled genomes are provided in A2 in S1 Data. Based on CheckM quality assessments, all genomes had <2% contamination and were estimated to be at least 99% complete except for isolate PS807 which had an estimated completeness of 44.27% (A2 in S1 Data). This latter isolate was only included in phylogenomic analyses but not in comparative genomics studies. Phylogenomic analyses of pseudomonad bacteria from almond tissues identified a high diversity of *Pseudomonas* species (A3 and A5 in S1 Data). Core genome-based approximate maximum likelihood phylogenomic analyses resulted in the isolates clustering into four main clades or phylogenomic groups (Fig 1). The main clades were designated as *P. syringae* species complex (clade I), *P. fluorescens* species complex (clade II), *P. simiae* species complex (clade III), and *Pseudomonas A* species complex (clade IV) (Fig 1). Clade IV contained no established *Pseudomonas* spp. (A2 in S1 Data), and therefore we designated this clade as *Pseudomonas* A species complex. Taxonomic assignment of the isolates was based on dDDH, and ANIb, which were in congruence (A3 and A4 in S1 Data). Whole genome sequencing-based phylogenomic analyses greatly improves the identification of isolates through the elucidation of functional profiles of taxonomic groups, and this can resolve ambiguities in the phylogeny of higher taxa, that would be difficult using traditional approaches [8, 9, 50]. Of the four designated clades, only the *P. syringae* species complex clade is known to include plant pathogenic bacteria. The *P. syringae* species complex also includes non-pathogenic isolates that occur naturally as epiphytes on plants and in the environment [14, 51]. While non-pathogenic *P. syringae* isolates were also identified in this study, our analyses identified three species that were pathogenic to almond. These are *P. syringae* pv. *syringae* (18 isolates), *P. cerasi* (2 isolates), and *P. viridiflava* (4 isolates), (Fig 2). Thus, 75% of the almond-pathogenic isolates in this study were *P. syringae* pv. *syringae*. This confirms *P. syringae* pv. *syringae* as the main causal agent of bacterial blast and bacterial canker of almond in California. *P. cerasi* was solely isolated from cankers and only represented 8.3% (2 isolates) of the total number of isolates pathogenic to almond. *P. viridiflava* isolates composed 16.6% of the pathogenic isolates and were recovered from symptomatic and asymptomatic tissues. The designated *P. syringae* species complex contained 38 isolates, *P. syringae* pv. *syringae* isolates composed 47.4% whereas *P. cerasi* and *P. viridiflava* isolates composed only 5.3 and 10.5%, respectively, of the isolates in this complex (Fig 2). Members of the *P. syringae* species complex has been divided into 13 phylogroups (PG) based on multi-locus sequence analyses [13]. Our isolates grouped into three established phylogroups: PG2 (*P. syringae* pv. *syringae* and *P. cerasi*), PG7 (*P. viridiflava*), and PG13 (*Pseudomonas spp*.) (S1 Fig). Isolates in PG13 clustered with *P. syringae* UB246 and could not be assigned to any established species, perhaps suggesting that they are a new species. Gomila et al. [52] assigned *P. syringae* UB246 to a putative new species and suggested that more closely related strains are needed for a definitive taxonomic assignment. Isolates from our study could be used for this, and our data indicate that this putative new species also can be found on almond. Several isolates were phylogenetically placed within the *P. syringae* species complex phylogenomic group/clade albeit they were not assigned to any specific phylogroup (S1 Fig). This shows how diverse the members of the *P. syringae* species complex are, thereby posing a challenge for classification, particularly when using low-resolution taxonomic methods. This signifies the importance of whole genome-based phylogenomic analyses for taxonomic assignment of members of the *P. syringae* species complex. All isolates from our study that could not be distinguished from the model strain *P. syringae* pv. *syringae* B728a by dDDH and ANIb were named *P. syringae* pv. *syringae*. However, based on dDDH calculations from the TYGS, these isolates and the model strain (B728a) are not the same species as the *P. syringae* type strain, forming their own species cluster (A3 and A4 in S1 Data). This result is consistent with the recent study on clarification of taxonomic status within the *P. syringae* species group based on phylogenomic analyses [52]. To avoid confusion, we referred to these isolates as *P. syringae* pv. *syringae* throughout the manuscript.

### Analysis of pathogenicity related genomic features

**Fig 3:**
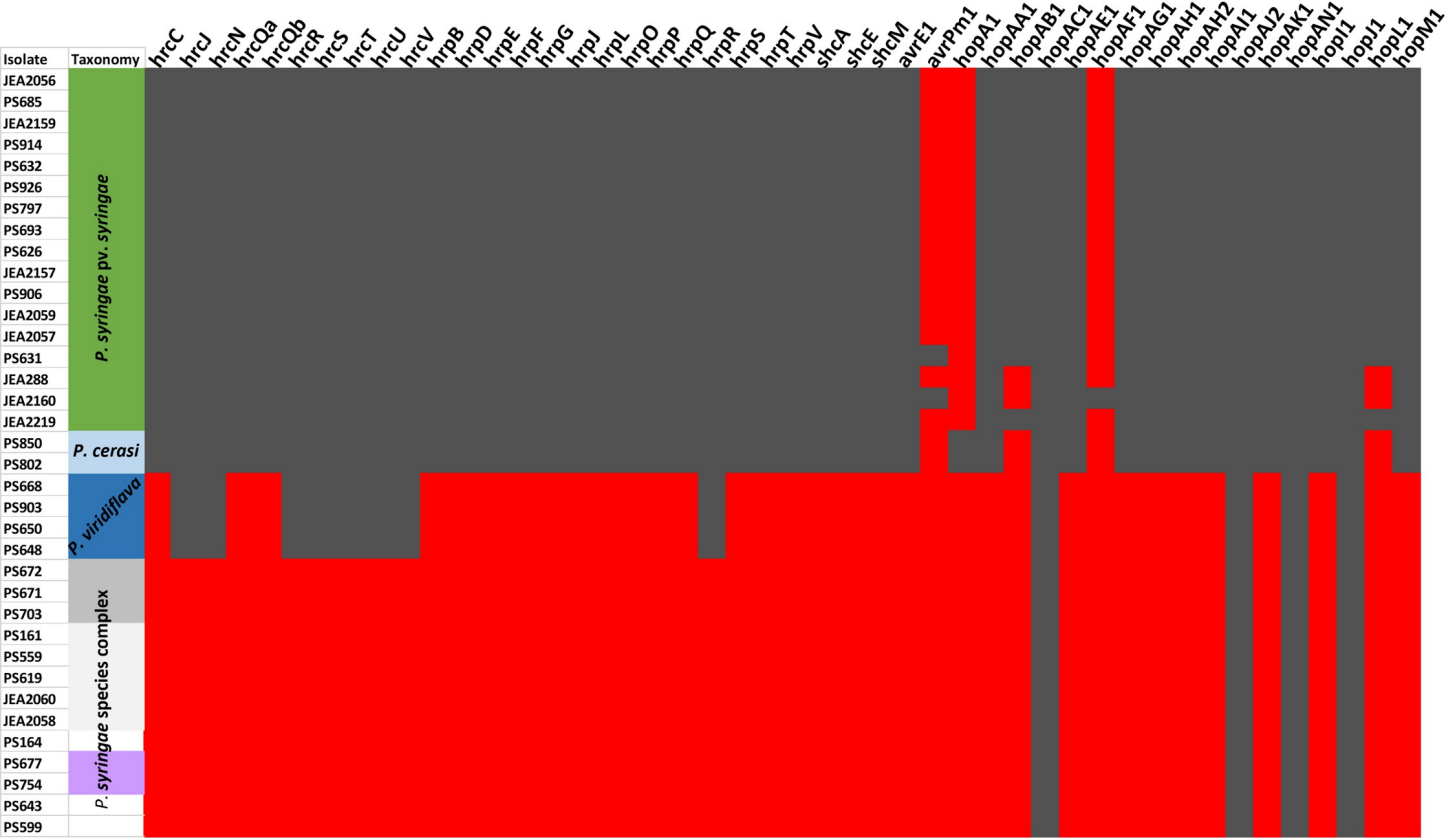
Annotated components of the type III secretion system (T3SS) from genomic sequences of *P. syringae* pv *syringae*, *P. cerasi*, *P. viridiflava* isolates and *P. syringae* isolates. Gray indicates that the corresponding T3SS component could be annotated from the genomic sequences, while red indicates that the component could not be annotated.

**Fig 4:**
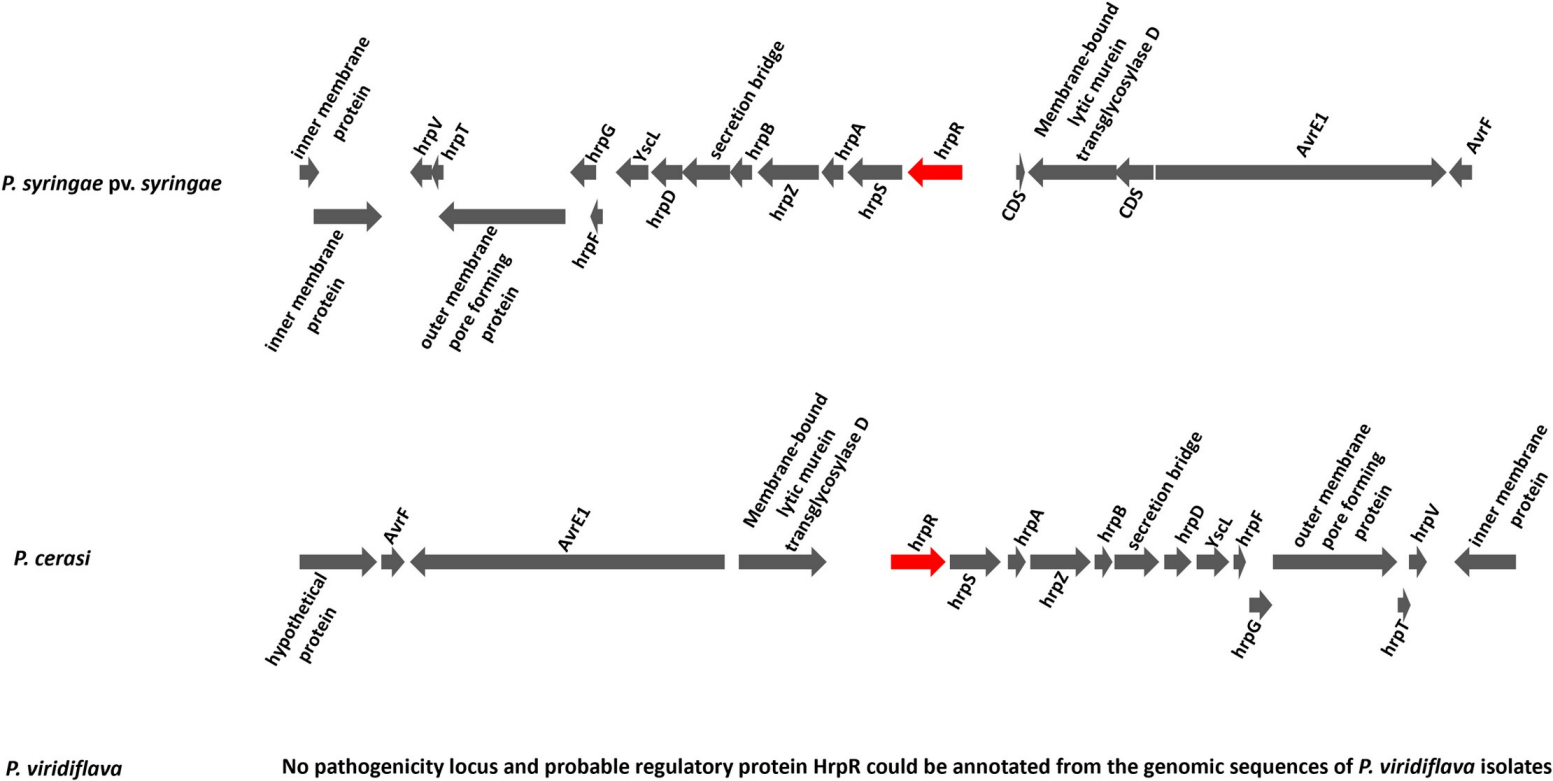
Structure of the annotated pathogenicity locus and probable regulatory protein HrpR from genomic sequences of *P. syringae* pv. *syringae* and *P. cerasi* isolates. Pathogenicity locus probable regulatory protein HrpR could not be annotated for all *P. viridiflava* isolates.

Pathogenic members of the *P. syringae* species complex carries a type III secretion system (T3SS), a key determinant for virulence [17, 18]. Genomic analyses of the three pathogenic species/pathovars revealed that they all harboured components of T3SS (Fig 3). Annotation of the genome sequences revealed genes encoding an intact pathogenicity locus, a probable regulatory protein HrpR, and the associated proteins in *P. syringae* pv. *syringae* and *P. cerasi* isolates (Fig 4). In contrast, the locus could not be identified in *P. viridiflava* genomic sequences. Fewer components of the *hrp/hrc* cluster were identified in *P. viridiflava* compared to *P. cerasi* and *P. syringae* pv. *syringae* (Fig 3). Such genomic variations in the architecture of the T3SS are common among (and sometimes within) the *P. syringae* species complex phylogroups [6, 30, 53]. This is attributed to the members of the species complex being apt to survive in a wide range of habitats, and such environmentally driven heterogeneity in bacteria has been described in many studies [6, 54]. Besides *P. syringae* pv. *syringae*, *P. cerasi*, and *P. viridiflava*, other almond isolates in the *P. syringae* species complex had no annotated *hrp/hrc* components (Fig 3). There was also variation in *avr* and *hop* genes with *P. syringae* pv. *syringae* and *P. cerasi* having the most components. Unlike the *hrp/hrc* cluster, the *P. viridiflava avr* and *hop* clusters were like those of non-pathogenic *P. syringae* isolates (Fig 3). In addition, the genomes of *P. syringae* pv. *syringae* and *P. cerasi* had an ice nucleation gene, which correlated with the tested ice nucleation phenotype (Fig 2). In contrast, all other isolates classified within the *P. syringae* species phylogenomic group had no ice nucleation gene and did not exhibit ice nucleation activity (Fig 2). Bacterial ice nucleation is associated with the ability to cause frost damage that may lead to water and nutrient release from plants and could create openings on the plant surface to facilitate bacterial entry [23, 49].

**Fig 5:**
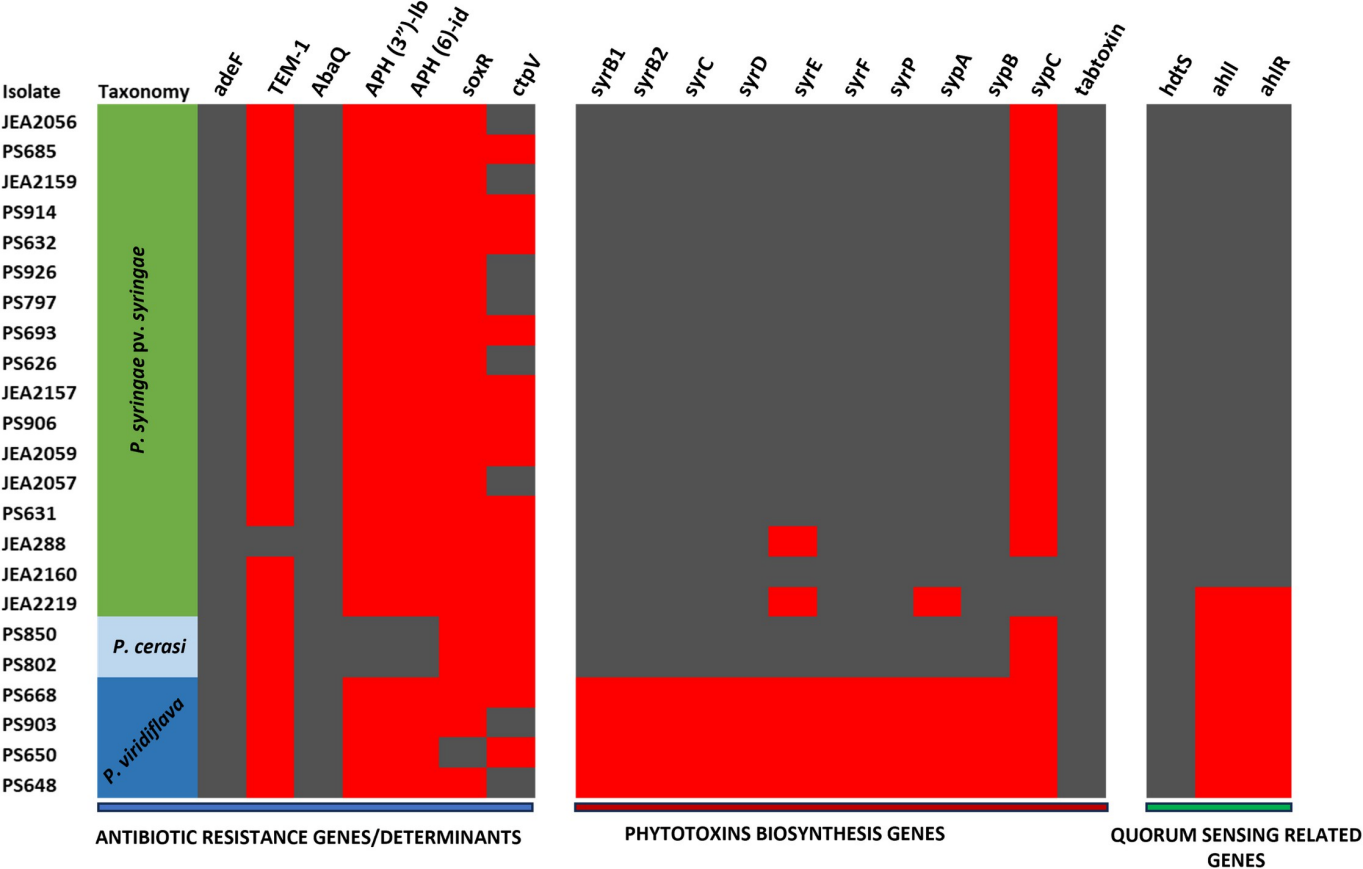
Genes for antibiotic resistance, phytotoxin biosynthesis, and quorum sensing annotated from the genomic sequences of *P. syringae* pv. *syringae*, *P. cerasi*, and *P. viridiflava*. Left: antibiotic resistance genes; Center: phytotoxin biosynthesis genes; Right: quorum sensing-related genes. Gray indicates that the corresponding gene could be annotated from the genomic sequences, while red indicates that the gene could not be annotated from the genomic sequences.

We also identified genes encoding phytotoxins known to be important for the virulence of members of the *P. syringae* species complex. *P. syringae* pv. *syringae* and *P. cerasi* isolates had genes that encode for the production of the phytotoxins syringopeptin, syringomycin, and tabtoxin (Fig 5). Syringomycin and syringopeptin cause membrane disruption in host plants, leading to ion leakage and the release of metabolites and act as biosurfactants, that subsequently increase wetness on the plant’s surface, facilitating easy bacterial movement [30, 55, 56]. In contrast, only genes encoding tabtoxins were annotated from the genomic sequences of *P. viridiflava* isolates (Fig 5). Biosynthetic gene clusters encoding for tabtoxin, were also annotated in all the other members of the designated *P. syringae* species complex. *P. viridiflava* isolates had large differences from other members of the *P. syringae* species complex in average nucleotide identity, virulence genes, and the core genome in general [5, 53, 57]. Moreover, *P. viridiflava* is the only member of the *P. syringae* species complex known to cause soft rot in plant tissue [58], and this is mainly attributed to the variation in virulence factors [57]. Thus, *P. viridiflava* is an entirely distinct phytopathogen in comparison with *P. syringae* pv. *syringae* and *P. cerasi*.

### Pathogenicity tests

**Fig 6:**
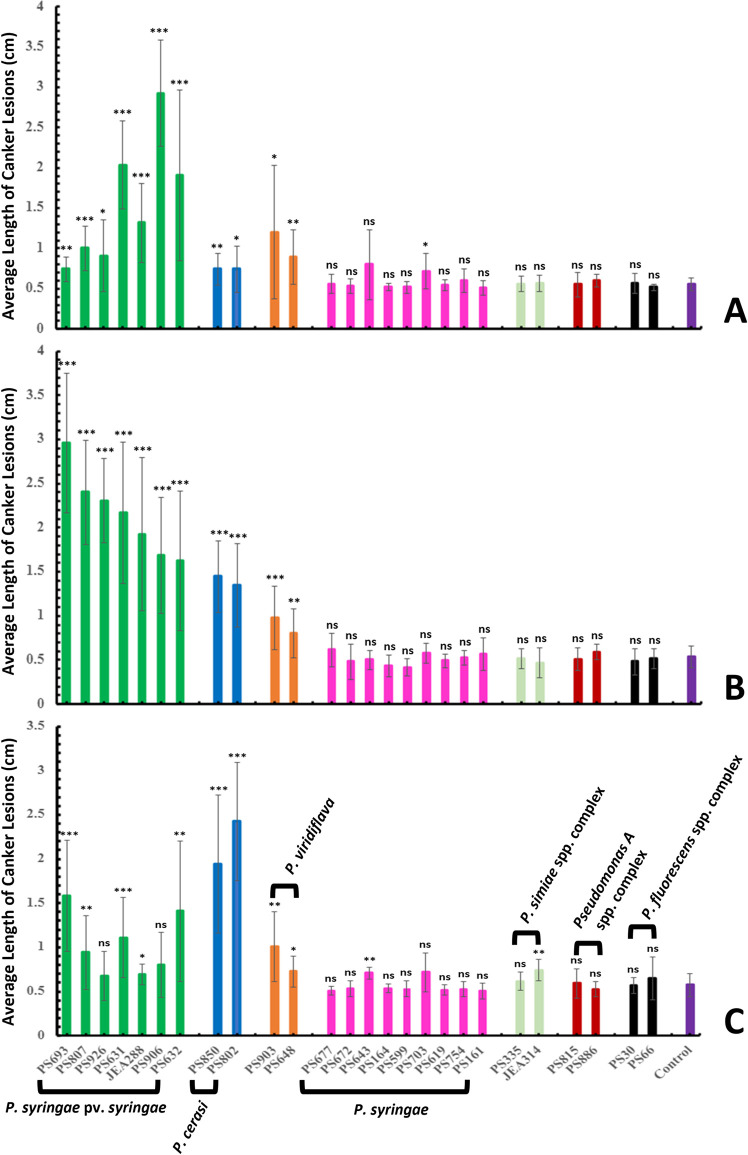
Canker pathogenicity tests in **A**. Nov 2022, **B**. Feb 2023, and **C**. Apr 2023. Data are the average of all replications (tree replicates), and error bars indicate standard deviation. *, **, and *** indicate statistical significance from the control with *P ≤ 0*.*05*, *P ≤ 0*.*01*, and *P ≤ 0*.*001*, respectively. ns indicates not statistically different from the control *P > 0*.*05*.

The pathogenicity of 26 representative isolates selected from all four designated species complexes was investigated in an almond orchard at Kearney Agricultural Research and Extension Centre (KARE). In the canker experiment conducted in November 2022, the results indicated that the most virulent strains were *P. syringae* pv. *syringae* (Fig 6A). *P. syringae* pv. *syringae* isolate PS906 caused the largest canker lesions (2.93 ± 0.66 cm) (Fig 6A). *P. syringae* pv. *syringae* isolates PS632, JEA288, and PS631 caused canker lesions ranging from 1.32 ± 0.49 to 2.03 ± 0.54 cm (Fig 6A). The two *P. cerasi* isolates PS802 and PS850 incited canker lesions 0.74 ± 0.19 and 0.74 ± 0.29 cm in size, respectively. *P. viridiflava* isolates PS903 and PS648 caused canker lesions of 1.2 ± 0.83 and 0.89 ± 0.33 cm, respectively (Fig 6A). Control treatment had canker lesions 0.56 ± 0.08 cm in size (Fig 6A). The lesions of all *P. syringae* pv. *syringae*, *P. cerasi*, and *P. viridiflava* isolates were significantly longer (*P ≤* 0.05) than those of the control (Fig 6A). Moreover, *P. syringae* isolate PS703 caused canker lesions of 0.72 ± 0.22 cm that were significantly longer (*P =* 0.0289) as compared to the control with 0.56 ± 0.08 cm (Fig 6A). In contrast, the canker lesions for representative isolates of all other designated species complexes were not statistically different from the control (Fig 6A). Although *P. syringae* isolate PS703, *P. cerasi* isolates PS802 and PS850, *P. viridiflava* isolate PS648, and *P. syringae* pv. *syringae* isolates PS693 and PS926 resulted in statistically larger lesions than the control, they appeared to be weak pathogens in this experiment. There were some notable variations in the degree of virulence among isolates of *P. syringae* pv. *syring*ae (Fig 6A). This is commonly observed among *P. syringae* pv. *syringae* isolates [59] and can be attributed to factors such as slight genetic variability and adaptations to the host plant [60]. Since all the isolates from this study originated from almond (except isolate PS807 which was isolated from cherry), we can presume that they are all almond-adapted. Thus, slight genetic variations might explain the differences in virulence, and this variation could be supported by the observed shared/unique orthologous genes among the isolates of *P. syringae* pv. *syringae* (S2 Fig). Although no unique orthologous genes had a pathogenesis gene ontology (GO) annotation, categories that could be involved in fitness, like transport, catabolic processes, and copper export, were among the unique orthologous genes.

For the experiment conducted in February 2023, the average length of canker lesions of all the tested *P. syringae* pv. *syringae* isolates ranged from 1.63 ± 0.79 cm to 2.96 ± 0.79 cm (Fig 6B). In contrast to the experiment conducted in November 2022, *P. syringae* pv. *syringae* isolates PS693 and PS807 were the most aggressive with canker lesion lengths of 2.96 ± 0.79 and 2.4 ± 0.59 cm, respectively (Fig 6B). PS807 was the only isolate in this study not originating from almond, although it was relatively higher virulent, suggesting its lack of host specificity. This is not surprising because isolates belonging to the *P. syringae* species complex have been shown to infect a wide range of plants [5]. Compared to the November 2022 experiment, *P. syringae* pv. *syringae* isolate PS906 appeared to have only moderate aggressiveness with canker lesions of 1.68 ± 0.66 cm in contrast to 2.93 ± 0.66 cm in the November experiment (Fig 6A and 6B). The two *P. cerasi* isolates were moderately aggressive, having average lesion lengths of 1.45 ± 0.41 cm and 1.35 ± 0.47 cm (Fig 6B). This was different from the experiment conducted in November 2022, where *P. cerasi* isolates appeared to be not pathogenic. The two *P. viridiflava* isolates had canker lesions of 0.975 ± 0.36 cm and 0.8 ± 0.28 cm (Fig 6B). Control cankers were 0.53 ± 0.12 cm long (Fig 6B). As in the experiment conducted in November, all isolates of *P. syringae* pv. syringae, *P. cerasi*, and *P. viridiflava* had canker lesion lengths that were significantly longer (*P ≤* 0.05) than those of the control (Fig 6A and 6B). All other isolates, which include isolates of *P. syringae*, and representative isolates of the species complexes *P. fluorescens*, *P. simiae*, and *Pseudomonas A* produced lesions that were not statistically different in size from the control (Fig 6B). Unlike the November experiment, canker lesion sizes for *P. syringae* isolate PS703 were not statistically different from the control in this experiment (Fig 6B).

In the experiment conducted in April 2023, *P. cerasi* isolates were the most aggressive (Fig 6C). Isolates PS850 and PS802 caused canker lesions 1.94 ± 0.76 and 2.43 ± 0.67 cm in length, respectively (Fig 6C). In contrast, the largest canker lesions for *P. syringae* pv. *syringae* (isolates PS632 and PS693) were 1.41 ± 0.79 and 1.58 ± 0.63 cm, respectively (Fig 6C). All isolates of *P. cerasi* and *P. viridiflava* as well as *P. syringae* pv. *syringae* isolates PS693, PS807, PS631, JEA288, and PS632 had significantly (*P ≤* 0.05) longer canker lesions than the control with 0.575 ± 0.13 cm (Fig 6C) but isolates PS926 and PS906 of the latter species were not statistically different from the control (Fig 6C). Moreover, *P. syringae* isolate PS643 and a representative isolate (isolate JEA314) of the *P. simiae* species complex had canker lesions significantly longer (*P ≤* 0.05) than the control (Fig 6C). In all the experiments, the inoculated isolates were recoverable from canker margins, and their identity was confirmed using PCR, thereby fulfilling Koch’s postulates. The observed variations in the November, February, and April experiments can be attributed to environmental conditions, and temperature in particular, during the period when the experiments were conducted. Members of the *P. syringae* species complex has been shown to exhibit a variable temperature-dependent expression of virulence factors [61–63]. Thus, the April weather conditions might have been more conducive for the expression of *P. cerasi* virulence determinants compared to the other species.

**Fig 7:**
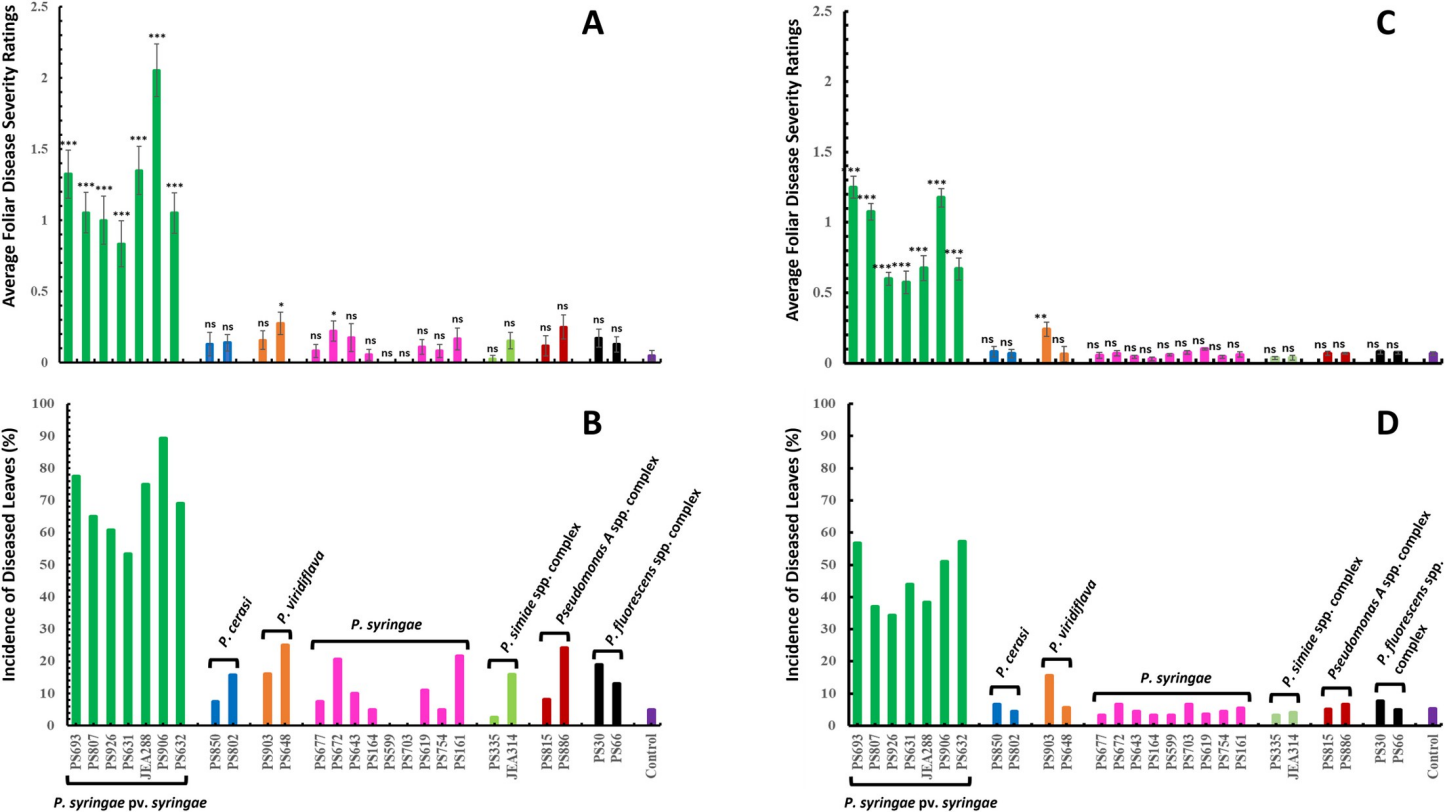
Results of leaf pathogenicity tests. (A) Average severity and (B) Average disease incidence of the first experiment; and (C) Average severity and (D) Average disease incidence of the repeat experiment for all isolates. Error bars indicate the standard errors. A severity score of 0 was assigned to intact leaves (no visible necrosis and spots), a score of 1 was assigned to leaves with visible marginal necrosis, a score of 2 was assigned to leaves with visible marginal necrosis having leaf spots covering less than 50% of the leaf blade, and a score of 3, was assigned to leaves with visible marginal necrosis and multiple leaf spots covering at least 50% of the leaf blades.

In pathogenicity tests using leaves in the field (blast phase), *P. syringae* pv. *syringae* isolates were the most aggressive, and they were the only strains capable of causing severe foliar symptoms (Fig 7A–7C). *P. syringae* pv. *syringae* isolates had average disease severity ratings ranging from 0.83 ± 0.96 to 2.05 ± 1.1 (Fig 7A). Control treatment had an average severity rating of only 0.05 ± 0.23 (Fig 7A). Foliar symptoms produced by all *P. syringae* pv. *syringae* isolates were significantly severe (*P ≤* 0.001) than that of the control (Fig 7A). In contrast, *P. viridiflava* and *P. cerasi* isolates incited average disease severity ratings of ≤ 0.28 (Fig 7A). Disease severity caused by all isolates of *P. cerasi* and that for *P. viridiflava* isolate PS903 were not statistically different from the control. Since the severity scores of *P. viridiflava* and *P. cerasi* isolates were not significantly distinct from the control (Fig 7A), we considered them non-pathogenic to almond leaves. Although the ratings for *P. viridiflava* isolate PS648 were statistically higher (*P =* 0.0118) than the control, the observed symptoms were not clearly distinct from the symptoms observed on the control treatment. All other isolates of *P. syringae* and representative isolates of *P. fluorescens*, *P. simiae*, and *Pseudomonas A* species complex had symptom severity ratings that were not statistically different except for *P. syringae* isolate PS672 (0.22 ± 0.42) that was statistically different (*P =* 0.0226) from the control (Fig 7A).

To more completely assess the ability of isolates to cause foliar disease symptoms, we also evaluated the incidence of diseased leaves for each strain. *P. syringae* pv. *syringae* isolates caused a range of 53.3 to 89.4% of leaves to exhibit disease (Fig 7B). *P. cerasi* isolates seldom caused disease (15.7 and 7.5%) (Fig 7B) while *P. viridiflava* isolates caused disease in only 25 and 20.6% of the leaves. Although 25% disease incidence is not negligible, it is important to note that the control treatment exhibited only 5% disease incidence. The relatively high disease incidence in control trees and trees treated with non-pathogens is likely due to the presence of low populations of natural pathogenic *P. syringae* in the orchard. Moreover, *P. viridiflava* had been reported to cause disease in synergy with other microbes [64], and the almond leaf microbiome might harbor some of these microbes which augment *P. viridiflava’s* virulence. The mechanism underlying this synergism is unknown [64–66]. A repeat of the experiment produced results that showed a similar trend to the first experiment, only *P. syringae* pv. *syringae* isolates caused significant disease symptoms on leaves (Fig 7C). All *P. syringae* pv. *syringae* isolates had disease severity ratings statistically greater (*P ≤* 0.001) than the control (Fig 7C). In contrast, no other isolates except for *P. viridiflava* isolate PS903 had symptom ratings that were significantly different (*P ≤* 0.05) from the control (Fig 7C). Moreover, disease incidence ratings for *P. syringae* pv. *syringae* isolates ranged from 34.4 to 67.2% (Fig 7D). In contrast, disease incidence ratings for all the other isolates were ≤ 15.6% (Fig 7D). Disease incidence rating for the control was 5.3% (Fig 7D).

**Fig 8:**
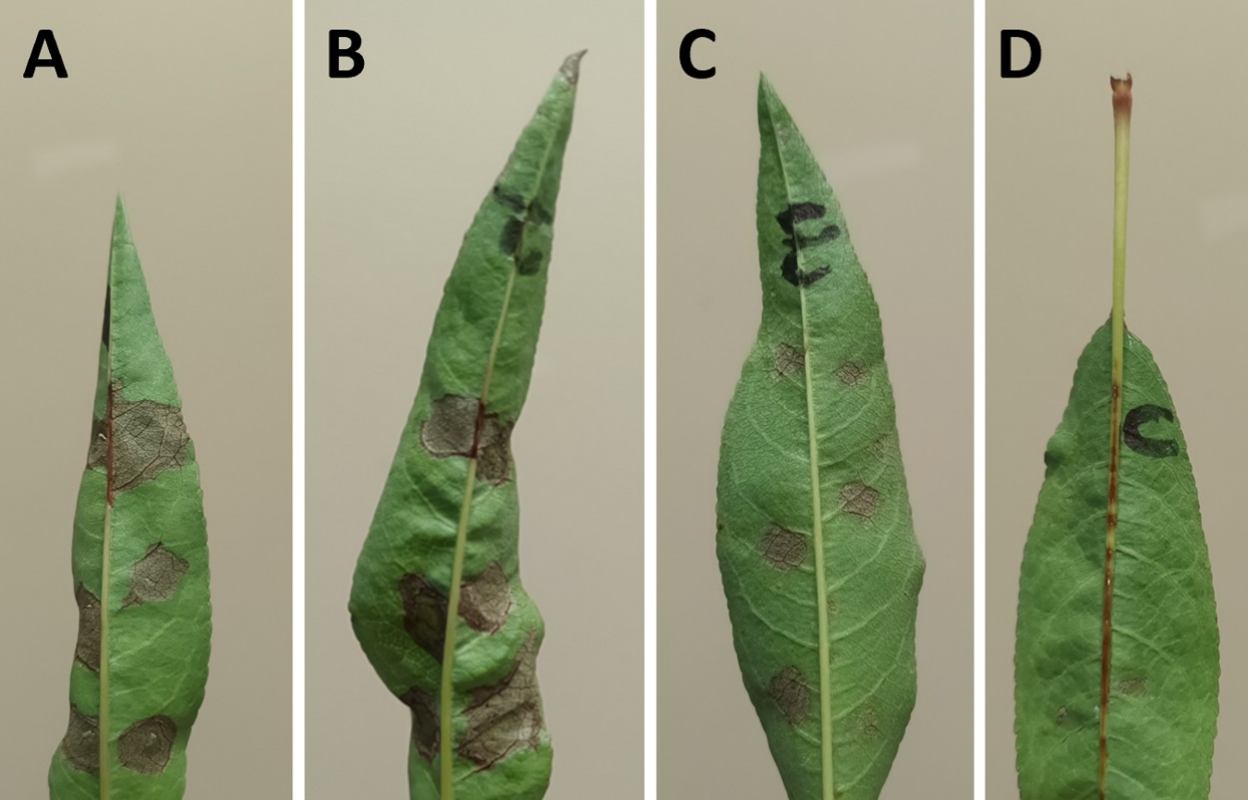
Results of detached leaf pathogenicity bioassay. (A) almond leaf inoculated with *P. syringae* pv. *syringae*. (B) almond leaf inoculated with *P. cerasi*. (C) almond leaf inoculated with *P. viridiflava*. (D) control, almond leaf inoculated with PBS.

In further attempts to understand the pathogenicity of *P. cerasi* and *P. viridiflava* isolates on almond leaves, we conducted detached leaf bioassays. In contrast to the field leaf pathogenicity tests, *P. syringae* pv. s*yringae* and *P. cerasi* strains were very aggressive towards detached almond leaves (Fig 8). Both *P. cerasi* and *P. syringae* pv. *syringa*e caused necrosis/browning that was ≥ 0.7 cm on detached almond leaves (Fig 8). In contrast, *P. viridiflava* caused some browning/necrosis on detached almond leaf assay albeit it was lower than that caused by *P. cerasi* and *P. syringae* pv. *syringae*, causing lesions < 0.5 cm (Fig 8). Control treatments had no visible necrosis/browning, an indication that both *P. viridiflava* and *P. cerasi* can infect the almond leaves (Fig 8). This result indicates that *P. cerasi* can cause severe symptoms on leaves however, it is probably a weak epiphyte and cannot withstand the hostile leaf surface environment presented in the field experiments. Quorum sensing positively regulates several traits that are crucial for epiphytic fitness, and cells in large aggregates are resistant to environmental stress to which they are exposed during the epiphytic phase [67, 68]. *N*-acyl homoserine lactone (AHL) is the quorum-sensing signal molecule in *P. syringae* pv. *syringae*, and its production requires the expression of the AHL synthase gene, *ahlI*, and the AHL regulator gene, *ahlR* [68, 69]. All *P. syringae* pv. *syringae* isolates from this study have *ahlI* and *ahlR* (Fig 5), but these genes were not detected in any of the *P. cerasi* and *P. viridiflava* isolates (Fig 5). AhlI mutants of *P. syringae* pv. *syringae* have been shown to have a lower ability to survive desiccation stress on leaves [70]. This lack of *ahlI* in *P. cerasi* isolates could explain their inability to cause severe foliar symptoms in the field experiments, since they may not survive the hostile leaf surface environment as well. AhlI mutants of *P. syringae* pv. *syringae* could be used in further investigations as controls to compare the pathogenicity with the wild-type *P. cerasi* and *P. viridiflava*. The ability of *P. cerasi* to cause severe symptoms on the laboratory leaf infiltration assay can be explained by assisted infiltration into the apoplast, as well as a conducive environment (100% humidity). The pathogenicity of strains belonging to the *P*. s*yringae* species complex mainly relies on the presence of the *hrp/hrc* gene cluster [71]. All *P. cerasi* and *P. syringae* pv. *syringae* isolates had an intact typical *hrp/hrc* gene cluster (Figs 3 and 4), explaining the symptoms observed in the leaf infiltration assays. This may also explain their ability to cause cankers after the bark was punctured with a nail creating an entry point for the bacteria. In contrast, an intact *hrp/hrc* cluster was not detected in *P. viridiflava* genomic sequences, explaining why it was not very aggressive even under a conducive environment (100% humidity).

All *P. cerasi* isolates had an ice nucleation gene that correlated with the ice nucleation phenotype (Fig 2). Bacterial ice nucleation facilitates frost injury to plants and potentially creates openings on the plant’s surface that facilitates bacterial entry [49]. Thus, although *P. cerasi* did not cause significant foliar disease symptoms in the field, it has the potential to do so under cold conditions. Though *P. viridiflava* isolate PS903 appeared to show modest virulence in canker pathogenicity tests and leaf infiltration bioassays, isolates of this species as a whole did not appear to be aggressive almond pathogens in our studies (Figs 4 and 5). *P. viridiflava* has generally been considered a weak or opportunistic pathogen and a poor competitor for resources within microbial communities [72]. Moreover, the lack of a complete T3SS and a reduced T3E repertoire in *P. viridiflava* has been linked to its lack of pathogenicity [3, 53]. The classical *P. syringae* pathogenicity locus and the regulatory protein HrpR were not present in any of the *P. viridiflava* isolates, and they had an extremely reduced complement of T3E (Fig 3). This could explain the observed pathogenicity phenotypes for the *P. viridiflava* isolates.

### Genotypic antibiotic resistance profile, kasugamycin and copper sensitivity tests

Genes conferring resistance to antibiotics/bactericides are important for the success of phytopathogens and other bacteria as they can help withstand environmental selective pressure [54]. Genome analyses revealed the presence of a gene encoding CtpV in some *P. syringae* pv. *syringae* and *P. viridiflava* but not in *P. cerasi* isolates (Fig 5). *P. cerasi* isolates instead had the genes *aph(3’)-IIb*, and *aph(6)-Id*, which are known to be involved in the inactivation of aminoglycoside antibiotics (Fig 5). These genes were not found in any of the *P. syringae* pv. *syringae* and *P. viridiflava* isolates. Genes encoding AdeF and AbaQ that confer resistance to oxytetracycline were present in all three pathogenic species/pathovars (Fig 5). Genes encoding TEM-1 that confers resistance to beta-lactam antibiotics was detected only in *P. syringae* pv. *syringae* isolate JEA288 (Fig 5). A7 in S1 Data provides detailed information of all detected antibiotic resistance genes and the group of drugs they confer resistance to.

**Fig 9:**
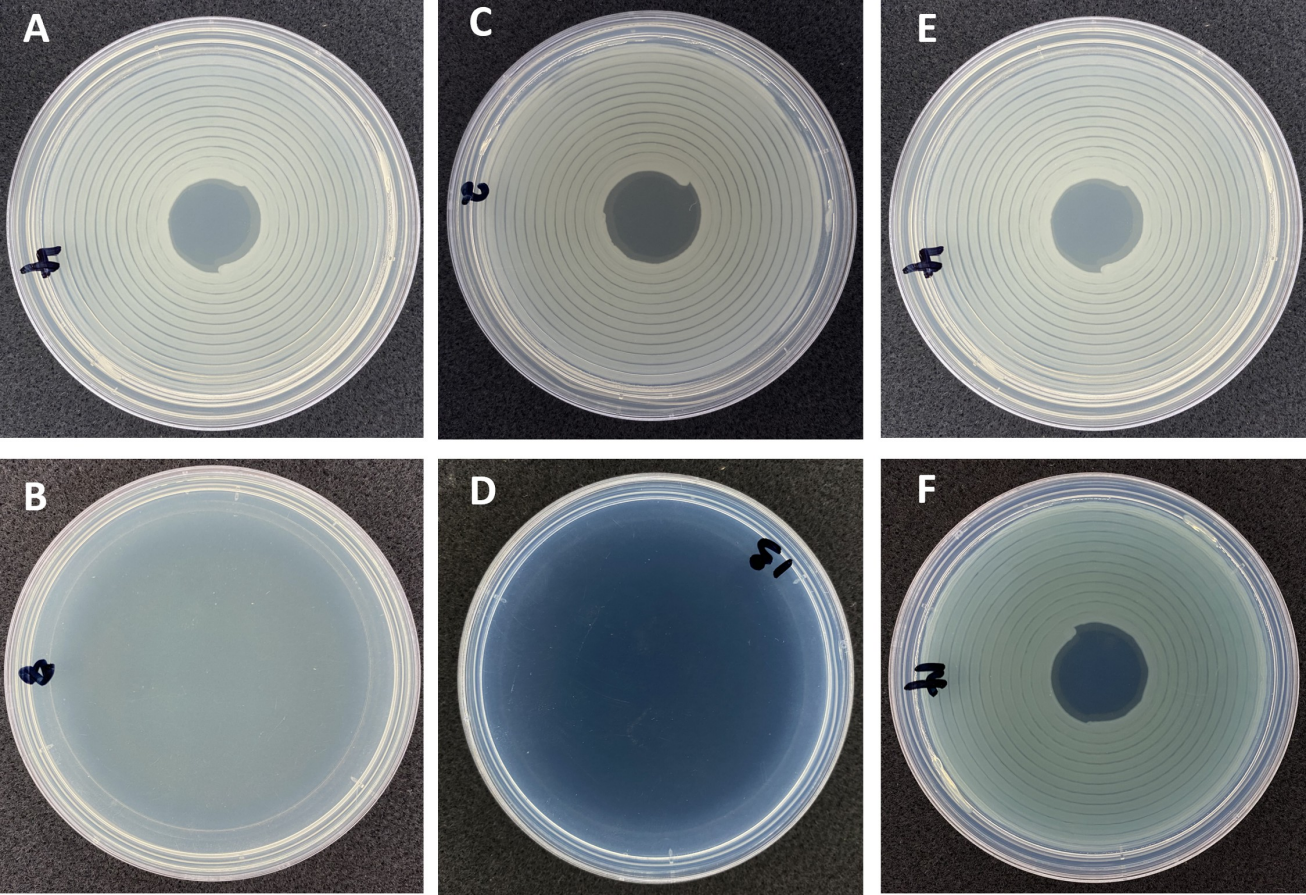
Representative antibiotic sensitivity tests. (A) control plate with visible bacterial growth. (B) plate amended with kasugamycin at 100 ppm, no visible bacterial growth indicating a susceptible phenotype; all isolates did not grow at 100 μg/ml kasugamycin. (C) control plate. (D) plate amended with 300 μg/ml metallic copper equivalent (MCE), no visible bacterial growth an indication of susceptible phenotype. (E) control plate (F) plate amended with 300 μg/ml MCE; visible growth indicates a resistant phenotype. All isolates with the *ctpV gene* grew on plates amended with up to 600 μg/ml MCE, in contrast, all isolates without the *ctpV* gene did not grow on plates amended with 200 μg/ml MCE.

Kasugamycin and copper are the two main bactericides used to control bacterial blast and bacterial canker of almond in California. Copper and kasugamycin sensitivity tests were done using six isolates of *P. syringae* pv. *syringae*, two isolates of *P. cerasi*, and four isolates of *P. viridiflava* to determine if resistance to these products is present. The isolates of *P. syringae* pv. *syringae* and *P. viridiflava* showed variation in sensitivity to copper. Three of the *P. syringae* pv. *syringae* isolates and two of the *P. viridiflava* isolates without the *ctpV* showed reduced growth in the presence of 200 μg/ml MCE, whereas three isolates of *P. syringae* pv. *syringae* and two isolates of *P. viridiflava* that had the gene grew on this amended medium (Fig 9). These latter isolates harboring c*tpV* also grew at 300, 400, 500, and 600 μg/ml MCE. In contrast, none of the *P. cerasi* isolates harbored c*tpV*, and they did not grow in the presence of 200 μg/ml MCE. CtpV is a putative copper exporter and is involved in the virulence of *Mycobacterium tuberculosis* [73]. The presence of CtpV in *P. syringae* pv. *syringae* and *P. viridiflava* appears to be responsible for their copper resistance phenotype. No other copper resistance genes were detected. These findings suggest that copper cannot be used to successfully manage bacterial blast and bacterial canker of almond, particularly in fields where *P. syringae* isolates have *ctpV*. The presence of c*tpV* in copper-resistant phenotypes suggests that this gene can be used to predict copper resistance in orchard samples. We thus designed primers to amplify c*tpV* (A7 in S1 Data).

All isolates did not grow in the presence of 100 μg/ml (Fig 9) or 75 μg/ml kasugamycin, suggesting that this antibiotic can be effectively used to manage bacterial blast and bacterial canker of almond. The label recommended rate for kasugamycin in almonds is 100 μg/ml. Aminoglycoside phosphotransferases (APH) constitute a family of enzymes that confer resistance to a wide spectrum of aminoglycosides [74]. Although aminoglycoside inactivation enzymes APH (3)-IIb and APH (6)-Id were found in *P. cerasi* isolates, kasugamycin has a unique structure [75], and there is no evidence that these determinants confer resistance to kasugamycin. Resistance to kasugamycin is conferred by altering the target methylated nucleotides in 16S rRNA and the disruption of the KsgA methyltransferase [76, 77]. In *P. syringae*, the KsgA methyltransferase is referred to as RsmA. Moreover, the novel gene *aac(2′)-IIa* encoding 2′-*N*-acetyltransferase can inactivate kasugamycin [78]. Inspection of the *rsmA* in the pathogenic isolates in this study revealed no evidence of alteration of the target nucleotides or disruption of the gene. In addition, *acc 2-lla* was not detected in any of the isolates. Periodical antibiotic resistance testing of isolates from orchards to monitor for resistance is prudent. Prolonged use of kasugamycin to control bacterial brown stripe of rice caused by *Acidovorax avenae* subsp. *avenae* in Japan has resulted in the emergence of resistant field isolates that has resulted in lack of disease control [79]. The emergence of resistant isolates emphasizes the need for resistance management, which can be delayed using bactericide rotations and limiting the total number of applications of kasugamycin per year. On almond in California, the bactericide should be used only when risk of bacterial blast and canker is high, as overuse could lead to the development of resistance. Table 1 provides a summary of results for all the tested phenotypes in this study.

**Table 1:** Summary of the tested phenotypes results.

Tested phenotypes	*P. syringae pv. syringae*	*P. cerasi*	*P. viridiflava*
Canker Pathogenicity (Field)	significant ^a^	significant ^a^	not significant
Leaf spots (Field)	significant ^b^	not significant	not significant
Lesions on detached leaves	Severe	severe	not severe
Ice Nucleation	100%	100%	0%
Kasugamycin Sensitivity Tests	100%	100%	100%
Copper Sensitivity Tests	66%	100%	50%

^a^ indicates that the group had statistically longer canker lesions in at least one experiment

^b^ indicates that the group had statistically more severe leaf spot ratings and at least 50% disease incidence in at least one experiment.

### Species-specific identification by qPCR

Primers were designed that target species-specific sequences in the genes encoding virulence factors AvrE in *P. cerasi* and *P. syringae* pv. *syringae*, as well as of HrpR in *P. viridiflava*. A7 in S1 Data provides the details for each designed primer. The product length of each of the target regions is ≤ 400 bp, making the primers suitable for use in qPCR. These primers were highly specific to the intended targets and can be used in endpoint and real-time PCR (A8 in S1 Data). The primers yielded linear amplifications over the range of ten-fold template dilutions with a correlation coefficient R^2^ = 0.998, indicating that the primers are highly efficient (A8 in S1 Data). Therefore, they can be used in population dynamics studies which could help in disease modelling and prediction of outbreaks.

## Conclusions

In this study, we established that *P. syringae* pv. *syringae*, *P. cerasi*, and *P. viridiflav*a are all almond pathogens, albeit *P. viridiflava* appears to be a weak pathogen. Since only two isolates were identified as *P. cerasi*, the frequency of isolation of *P. cerasi* in almond orchards was very low. Such low frequency of isolation could explain why *P. cerasi* was never considered an almond pathogen in California. Still, both isolates of *P. cerasi* were aggressive canker causing organisms, indicating that this species cannot be ignored as an almond pathogen. Only one of the *P. viridiflava* isolates was isolated from symptomatic tissue (canker), however, our pathogenicity tests showed that it is a very weak almond pathogen and does not pose a significant economic threat. *P. syringae* pv. *syringae* isolates were mainly from symptomatic tissues (canker, leaf lesions, and blasted flowers). *P. syringae* pv. *syringae* isolates were also aggressive in both canker and blast pathogenicity tests indicating that this organism is the main almond pathogen in California. A *P. syringae* pv. *syringae* isolate from cherry (PS807) was also aggressive in almond pathogenicity tests suggesting it can cause disease in more than one host. In California some almond orchards are adjacent to cherry orchards, and the pathogens can be easily moved from cherry to almond orchards and vice versa. This could also be a way in which *P. cerasi* could be introduced to almond orchards, assuming it is preferentially a cherry pathogen [80, 81]. Several cherry samples have recently tested positive for *P. cerasi* in our labs. Although *P. cerasi* samples from this study constituted only 8.3% of the pathogenic isolates, they were aggressive on almond, and this species is abundant in California sweet cherry orchards based on preliminary observations. Besides causing large cankers, *P. cerasi* isolates were aggressive in detached leaf bioassays but did not cause significant leaf symptoms in the field. This could be partly explained by lack of quorum sensing-related genes which significantly correlates with epiphytic fitness. However, *P. syringae* pv. *syringae* and *P. cerasi* isolates harbored the ice nucleation protein and were able to catalyze ice formation at warm subfreezing temperatures. This indicates that they are capable of causing frost damage to plants at temperatures slightly below 0°C and steps to reduce frost damage should be taken particularly if these populations are detected in an orchard during winter. Genotypic and phenotypic data indicated that all pathogenic isolates from our collection were susceptible to kasugamycin; thus, kasugamycin can be effectively used under the current emergency registration to manage bacterial blast and bacterial canker of almonds in California. However, kasugamycin efficacy is reduced by photodegradation of the molecule [82], and growers need to consider this by avoiding application during times of the day with higher light intensity. Resistance to copper is widespread in California orchards, and growers should test for resistance before relying on copper for managing bacterial blast and bacterial canker of almond. Primers targeting c*tpV* should prove useful in such monitoring. Rotating between different bactericides and antibiotics will help slow the development of resistance to anyone compound. For example, in orchards where copper resistance is not detected, copper could be alternated with kasugamycin and growers should limit kasugamycin use to two applications per year. Periodical antibiotic resistance testing of isolates from orchards is also crucial to monitor for resistance. Periodical antibiotic resistance testing of orchard isolates should also be done, but the goal should be to develop additional bactericides to manage these bacterial diseases of almond and other *Prunus* spp.

## Supporting information

S1 Fig Classification of isolates belonging to the *P. syringae* species complex using the *P. syringae* established phylogroups. Isolates from almonds fall into three established phylogroups (PG); PG2: all *P. syringae* pv. *syringae* and *P. cerasi* isolates; PG7: all *P. viridiflava* isolates; PG13: putative new species with isolates PS677, PS754, PS653, and PS599. A red star indicates clade of isolates classified into the *P. syringae* species complex, but were not classified to an established PG.
(PDF)

S2 Fig Orthologous cluster analysis of *P. syringae* pv. *syringae* isolates used in pathogenicity tests. The UpSet table displays unique and shared orthologous clusters among the isolates. The left horizontal bar chart shows the number of orthologous clusters per isolate, while the right vertical bar chart shows the number of orthologous clusters shared among the isolates.
(PDF)

S1 Data (XLSX)
