# Supplementary material for: Phylogenomic analyses and comparative genomics of Pseudomonas syringae associated with almond (Prunus dulcis) in California
Source: PLoS One. 2024 Apr 11;19(4):e0297867. doi: 10.1371/journal.pone.0297867 (PMC11008872; doi:10.1371/journal.pone.0297867)
Supplement: S1 Fig — Isolates from almonds fall into three established phylogroups (PG); PG2: all P. syringae pv. syringae and P. cerasi isolates; PG7: all P. viridiflava isolates; PG13: putative new species with isolates PS677, PS754, PS653, and PS599. A red star indicates clade of isolates classified into the P. syringae species complex, but were not classified to an established PG. (PDF) [file pone.0297867.s001.pdf]

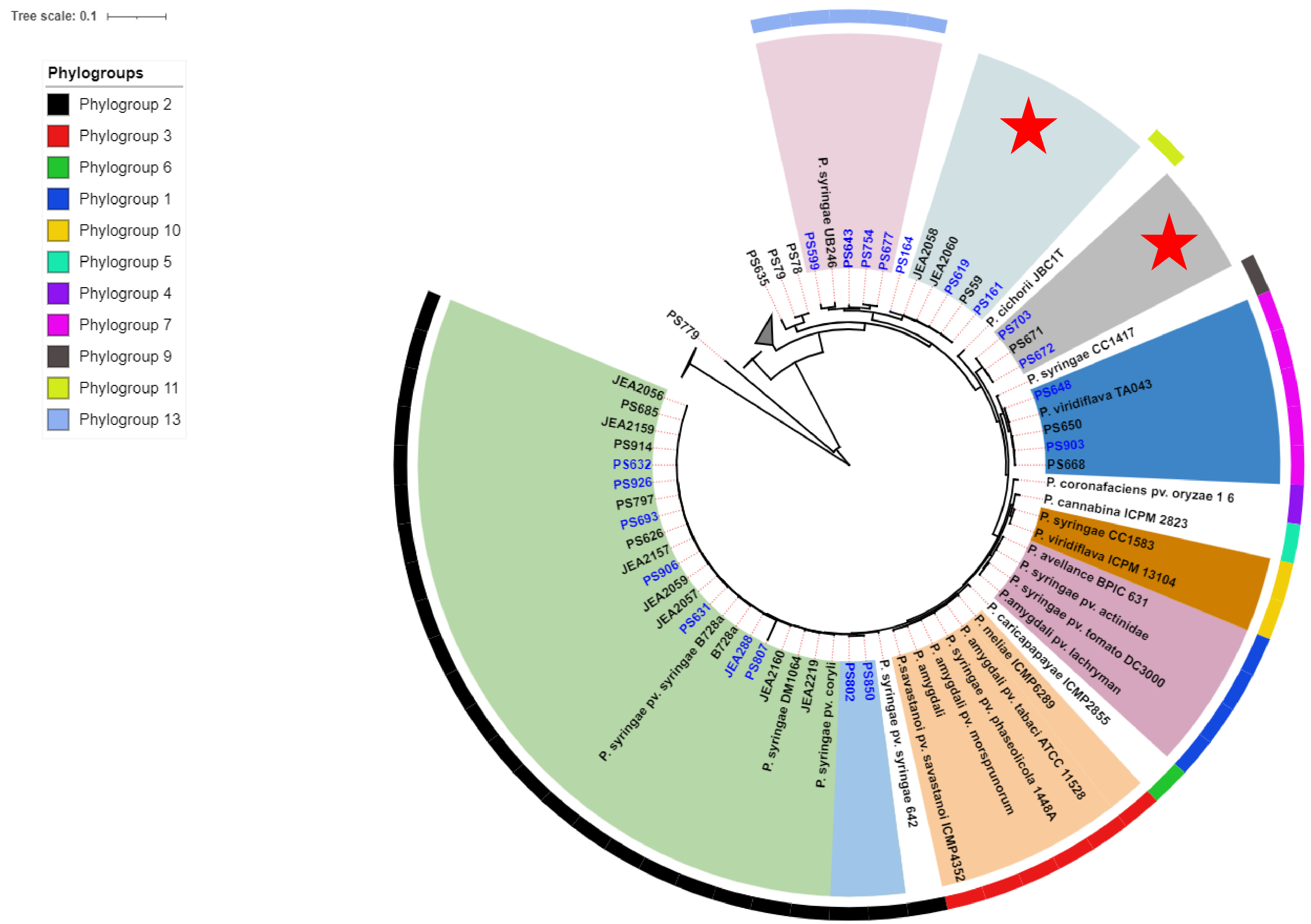
Tree scale: 0.1
Phylogroups
Phylogroup 2
Phylogroup 3
Phylogroup 6
Phylogroup 1
Phylogroup 10
Phylogroup 5
Phylogroup 4
Phylogroup 7
Phylogroup 9
Phylogroup 11
Phylogroup 13
PS779
PS635
PS79
PS78
PS599
P. syringae UB246
PS643
PS754
PS677
PS164
JEA2058
JEA2060
PS619
PS59
PS161
P. cichorii JBC1T
PS703
PS671
PS672
P. syringae CC1417
PS648
P. viridiflava TA043
PS650
PS903
PS668
P. coronafaciens pv. oryzae 1 6
P. cannabina ICPM 2823
P. syringae CC1583
P. viridiflava ICPM 13104
P. avellance BPIC 631
P. syringae pv. actinidae
P. syringae pv. tomato DC3000
P.amygdali pv. lachryman
P. caricapapayae ICMP2855
P. meliae ICMP6289
P. amygdali pv. tabaci ATCC 11528
P. syringae pv. phaseolicola 1448A
P. amygdali pv. morsprunorum
P. amygdali
P.savastanoi pv. savastanoi ICMP4352
P. syringae pv. syringae 642
PS850
PS802
P. syringae pv. coryli
JEA2219
P. syringae DM1064
JEA2160
PS807
JEA288
B728a
P. syringae pv. syringae B728a
PS631
JEA2057
JEA2059
PS906
JEA2157
PS626
PS693
PS797
PS926
PS632
PS914
JEA2159
PS685
JEA2056
