# Supplementary figures and images for: Phylogenomic analyses and comparative genomics of Pseudomonas syringae associated with almond (Prunus dulcis) in California
Source: PLoS One. 2024 Apr 11;19(4):e0297867. doi: 10.1371/journal.pone.0297867 (PMC11008872; doi:10.1371/journal.pone.0297867)

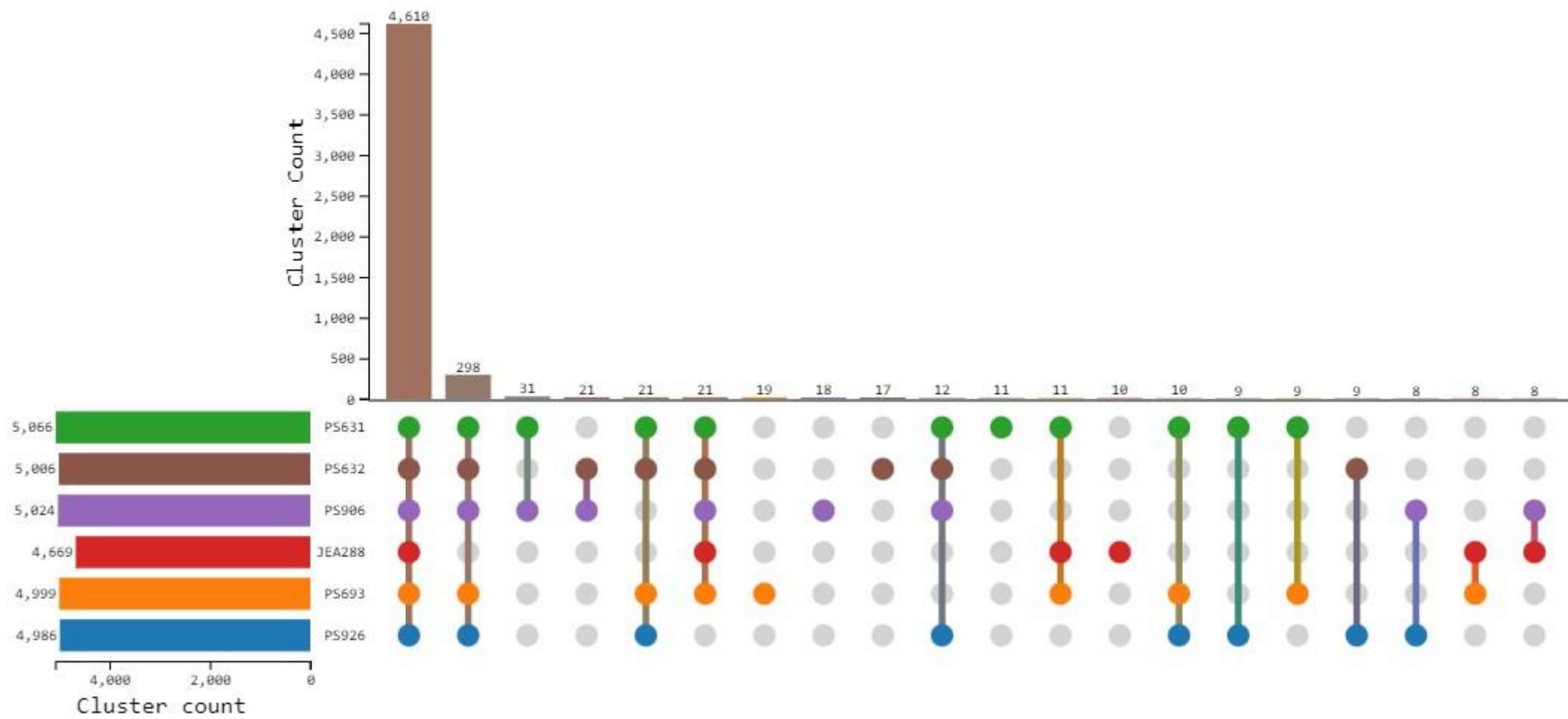

Supplement: S2 Fig — The UpSet table displays unique and shared orthologous clusters among the isolates. The left horizontal bar chart shows the number of orthologous clusters per isolate, while the right vertical bar chart shows the number of orthologous clusters shared among the isolates. (PDF) [file pone.0297867.s002.pdf]
